# Endoplasmic reticulum stress eIF2*α*–ATF4 pathway-mediated cyclooxygenase-2 induction regulates cadmium-induced autophagy in kidney

**DOI:** 10.1038/cddis.2016.78

**Published:** 2016-06-02

**Authors:** B Luo, Y Lin, S Jiang, L Huang, H Yao, Q Zhuang, R Zhao, H Liu, C He, Z Lin

**Affiliations:** 1 State Key Laboratory of Molecular Vaccinology and Molecular Diagnostics, School of Public Health, Xiamen University, Xiamen, China

## Abstract

The heavy metal cadmium (Cd) is nephrotoxic. Recent studies show that autophagy plays an essential role in Cd-induced kidney injury. However, the mechanisms of Cd-induced kidney injury accompanied by autophagy are still obscure. In the present study, we first confirmed that Cd induced kidney damage and dysfunction, along with autophagy, both *in vivo* and *in vitro*. Then, we observed that cyclooxygenase-2 (COX-2) and the eIF2*α*–ATF4 pathway of endoplasmic reticulum (ER) stress were induced by Cd in both kidney tissues and cultured cells. Further studies showed that inhibition of COX-2 with celecoxib or RNA interference (RNAi) inhibited the Cd-induced autophagy in kidney cells. In addition, blocking ER stress with 4-phenylbutyrate or RNAi partially counteracted COX-2 overexpression and autophagy induced by Cd, which suggested that ER stress was required for Cd-induced kidney autophagy. Significantly, our results showed that Cd activated ATF4 and induced its translocation to the nucleus. Knockdown of ATF4 inhibited Cd-induced COX-2 overexpression. While COX-2 overexpression is involved in renal dysfunction, there is no prior report on the role of COX-2 in autophagy regulation. The results of the current study suggest a novel molecular mechanism that the ER stress eIF2*α*–ATF4 pathway-mediated COX-2 overexpression contributes to Cd-induced kidney autophagy and injury. The present study implies that COX-2 may be a potential target for therapy against Cd-induced nephrotoxicity.

Cadmium (Cd) is a toxic heavy metal and ubiquitous environmental pollutant. Human exposure to Cd comes from several sources, such as metal industries, battery production, electroplating processes and cigarette smoking.^1, 2^ Cd exposure has serious detrimental effects on human health, such as bone softening, hepatic injury and renal dysfunction.^3, 4^ Previous studies showed that Cd-induced nephrotoxicity may occur through several mechanisms, such as oxidative stress,^5^ DNA damage,^6^ alteration of transcriptional regulation^7^ or apoptosis.^2, 8^ Toxic compounds usually kill cells via one of the three types of cell death: apoptosis, autophagy-dependent cell death and necrosis.^9, 10^ Recently, Cd has also been reported to induce autophagy in the kidney.^7, 10, 11^ As an adaptive response to stimuli, autophagy degrades macromolecules and organelles to maintain cell energy homeostasis and survival,^12^ but the molecular mechanisms of Cd-induced kidney autophagy are little understood.

Endoplasmic reticulum (ER), an organelle in cells, is involved in protein folding and trafficking and maintaining calcium homeostasis.^13^ When ER function is altered by intracellular or extracellular stimuli, ER stress occurs, with the accumulation of unfolded and misfolded proteins in the ER lumen, which activates an adaptive response commonly named the unfolded protein response (UPR). ER stress is transduced by three ER transmembrane proteins: protein kinase RNA-like endoplasmic reticulum kinase (PERK), inositol-requiring enzyme 1*α* (IRE1*α*) and activating transcription factor 6 (ATF6).^14^ Normally, the ER lumenal domains of the three transducers bind to glucose-regulated protein 78 (GRP78 or BiP). When UPR occurs, GRP78 prevents protein aggregation and helps them fold properly. As one major transducer of ER stress, PERK directly phosphorylates eukaryotic initiation factor 2 *α*-subunit (eIF2*α*), decreases most mRNA transcription and promotes transcription factor ATF4 translocation and activation. Accumulating evidence has indicated that ER stress triggers autophagy, which disposes of unfolded proteins in cells.^15, 16, 17^ However, there is no research exploring the role of ER stress in Cd-induced autophagy.

As the rate-limiting enzyme for prostaglandin E_2_ (PGE_2_) synthesis, COX-2 (encoded by the *PTGS2* gene) has been implicated in renal dysfunction and inflammation. Many lines of evidence indicate that COX-2 can be induced by Cd in several experimental models.^18, 19^ Nevertheless, there is no report on the role of COX-2 in autophagy induced by drugs or pollutants, especially Cd.

In the present study, we found Cd triggered autophagy accompanied by ER stress and COX-2 upregulation, both in mice kidney tissues and cultured human kidney cells. We hypothesized that ER stress and/or COX-2 would regulate Cd-induced kidney autophagy and wanted to provide insight into potential molecular mechanisms for intervention in Cd-induced nephrotoxicity. We showed, for the first time, that blocking COX-2 could inhibit Cd-induced autophagy. Furthermore, we found that Cd-induced COX-2 overexpression along with autophagy was inhibited by an ER stress inhibitor or RNAi. Notably, we revealed that Cd-induced kidney autophagy was mediated by COX-2 via eIF2*α*-ATF4 signaling transcriptional activation.

## Results

### Cd causes kidney injury both *in vivo* and *in vitro*

**Figure 1:**
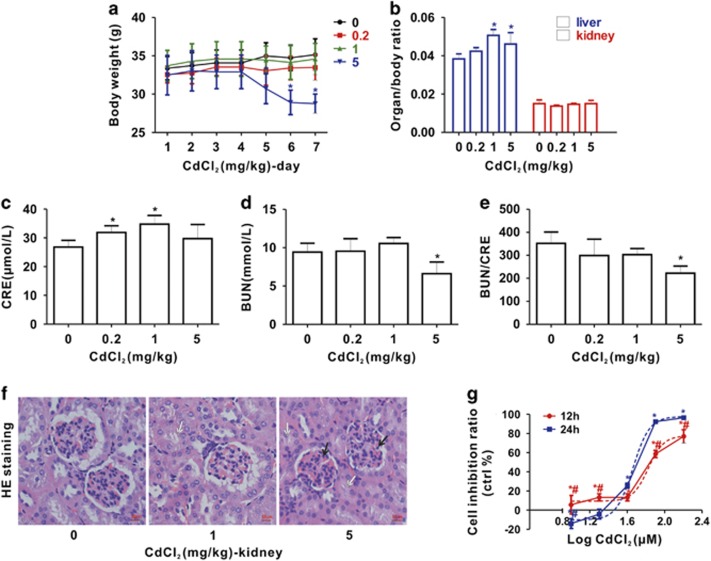
Cd causes kidney injury and cytotoxicity. (**a**) Body weight curve. (**b**) Ratios of liver and kidney weight to body weight. (**c**) CRE, (**d**) BUN and (**e**) BUN/CRE: renal function parameters; (**f**) histological changes (HE staining) of kidney after Cd exposure to mice for 7 days (magnification, × 400). Arrows indicate the histological changes of the glomerulus (black) and tubules (white). The scale bar is 50 *μ*m. (**g**) Cell growth of HEK cells using MTT assay. HEK cells are exposed to the indicated (log scale) Cd concentrations for 12 or 24 h, and the blue and red dotted lines stand for a nonlinear curve fitting as their IC_50_ values, respectively. (*Significantly different from untreated control and ^#^significantly different from different times at the same concentration. Data represent the mean±S.D.; (**a**–**f**), *n*=5–6; (**g**), *n*=3, *P<*0.05)

As shown in Figure 1a, the body weights of the 0.2 and 1 mg/kg Cd groups did not change compared with those of day 1, while those of the 5 mg/kg Cd group showed a significant decline from day 5 to day 7. We also calculated the ratios of liver and kidney to body weight and found that Cd significantly increased the ratio of liver to body in the 1 and 5 mg/kg Cd exposure groups, while there was no alteration in that of kidney to body weight (Figure 1b). Moreover, the mouse metallothionein (MT) genes (*mMT1* and *mMT2*) mRNA was significantly elevated both in livers and kidneys of mice exposed to Cd (Supplementary Figure S1a). Following this discovery, serum renal function parameters were analyzed. As shown in Figure 1c, we observed that serum creatinine (CRE) was raised in the 0.2 and 1 mg/kg Cd groups, but not changed in the 5 mg/kg group; serum blood urea nitrogen (BUN) was not significantly changed in the 0.2 and 1 mg/kg Cd groups, but significantly declined in the 5 mg/kg Cd group (Figure 1d); while the ratio of BUN/CRE was decreased in all three Cd groups, significantly in the 5 mg/kg Cd (Figure 1e). Further, histological changes were observed using hematoxylin and eosin (HE) staining. As shown in Figure 1f, shrinkage of glomeruli and the degeneration of tubules were observed in the 1 and 5 mg/kg Cd groups compared with the control group, which indicated that Cd damaged the kidney tissues, explaining the renal dysfunction demonstrated by the altered blood chemistry. These data indicated that renal function was damaged by Cd exposure *in vivo.*

*In vitro*, *hMT1B* increased in a time-dependent manner in HEK cells exposed to 40 *μ*M Cd (Supplementary Figure S1b). The MTT assay was used to evaluate cell growth after 12 and 24 h exposure to Cd (0–160 *μ*M). As shown in Figure 1g, cell growth decreased in a concentration- and time-dependent manner after Cd exposure compared with control (0.9% saline), and the 50% growth inhibition concentration (IC_50_) at 24 h was 51.6 *μ*M.

### Cd induces kidney cell autophagy both *in vivo* and *in vitro*

**Figure 2:**
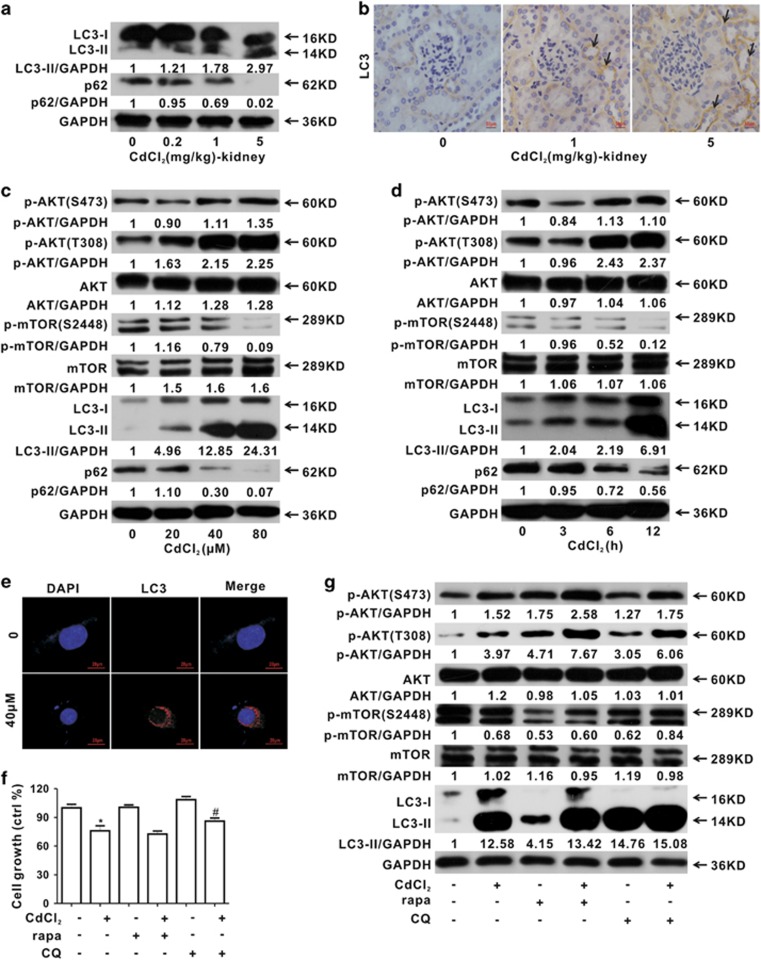
Cd induces kidney autophagy both *in vivo* and *in vitro*. (**a**) Autophagy-related proteins (LC3-II and p62) levels (treated with Cd for 7 days). (**b**) Immunohistochemical analysis for LC3 in kidneys of Cd-exposed mice (magnification, × 400). Arrows indicate the increase of LC3 in the tubules. Scale bar is 50 *μ*m. (**c** and **d**) Autophagy-related proteins (p-AKT, AKT, p-mTOR, mTOR, LC3 and p62) are analyzed using western blotting (**c**, as a function of Cd concentrations for 12 h; **d**, as a function of exposure time with 40 *μ*M Cd). (**e**) Immunofluorescence analysis for LC3 loci in Cd-exposed (40 *μ*M) HEK cells for 12 h. Scale bar is 20 *μ*m. (**f**) CQ, not rapa, enhances cell growth inhibited by Cd. (**g**) Rapa enhances autophagy induced by Cd (40 *μ*M) treatment for 12 h. (*Significantly different from untreated control, ^#^significantly different from Cd treatment. Data represent the mean±S.D.; *n*=3, *P<*0.05)

The major characteristics of autophagy are formation of autophagosomes and processing of LC3-I to LC3-II.^20^ In mice kidney tissues, LC3-II protein was sharply increased in all Cd exposure groups (Figure 2a). As a selective substrate of autophagy, p62 protein was significantly depleted (Figure 2a). Moreover, immunohistochemical analysis (Figure 2b) showed that LC3-II protein was significantly increased by Cd exposure in kidney tubules, which was quantitatively measured using Image Pro (Supplementary Figure S2). Furthermore, Cd exposure resulted in p-AKT elevation and p-mTOR decrease in HEK cells (Figures 2c and d). Along with these changes, the appearance of LC3-II and degradation of p62 increased in a concentration- and time-dependent manner after Cd treatment (Figures 2c and d). Next, the LC3-II loci were imaged after Cd exposure for 12 h using immunofluorescence analysis. As shown in Figure 2e, specific punctuate, LC3-II was observed as more dots visible in Cd-exposed cells than in control cells. To determine the role of autophagy in Cd-induced cytotoxicity, an autophagy inducer, rapamycin (rapa), and a lysosome inhibitor, chloroquine (CQ), were used. We found CQ, not rapa, rescued cell growth inhibition induced by Cd (Figure 2f). Moreover, rapa could enhance Cd-induced autophagy, as evidenced by the decrease of p-mTOR and increase of p-AKT and LC3-II (Figure 2g).

### Cd elevates COX-2 expression both *in vivo* and *in vitro*

**Figure 3:**
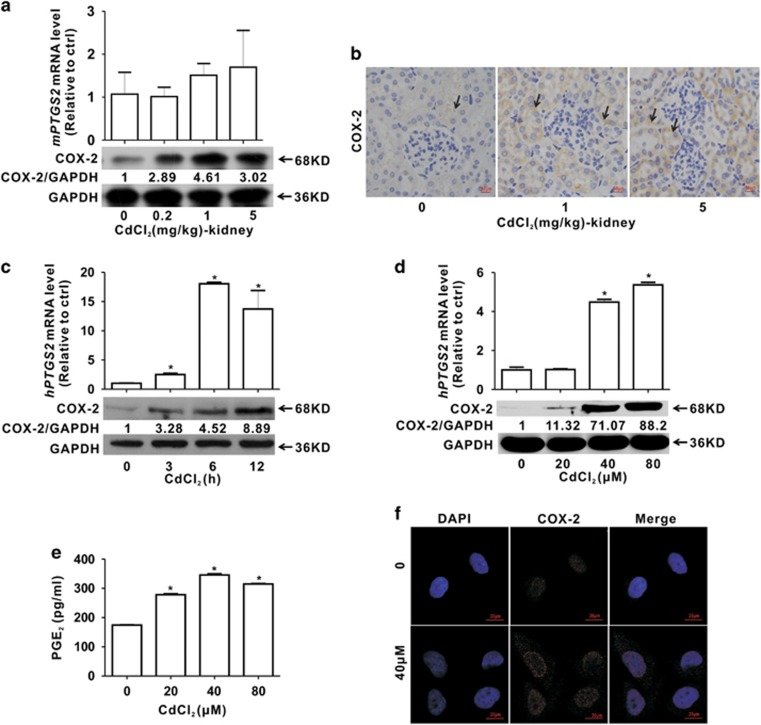
Cd upregulates COX-2 expression both *in vivo* and *in vitro*. (**a**) *mPTGS2* mRNA and COX-2 protein levels and (**b**) immunochemical analysis for COX-2 in the kidney tissues of Cd-exposed mice for 7 days (magnification, × 400). Arrows indicate the COX-2 signals in the kidney tubules. Scale bar is 50 *μ*m. (**c** and **d**) *hPTGS2* mRNA and COX-2 protein levels in HEK cells (**c**, as a function of exposure time with 40 *μ*M Cd; **d**, as a function of Cd concentrations for 12 h). (**e**) PGE_2_ production and (**f**) Immunofluorescence analysis for COX-2 are elevated in Cd-exposed HEK cells for 12 h (40 *μ*M). Scale bar is 20 *μ*m. (*significantly different from untreated control. Data represents the mean±S.D.; *n*=3, *P<*0.05)

To explore the role of COX-2 in Cd-induced renal dysfunction, COX-2 expression was assessed after Cd exposure. *In vivo*, as shown in Figure 3a, Cd increased *mPTGS2* mRNA and COX-2 protein levels in a dose-dependent manner. Furthermore, immunohistochemical analysis confirmed that COX-2 was significantly induced in kidney tubules by Cd exposure (Figure 3b and Supplementary Figure S2).

*In vitro* (in HEK cells), *hPTGS2* mRNA and COX-2 protein were significantly increased in a concentration- and time-dependent manner after Cd treatment (Figures 3c and d). PGE_2_ was also increased in Cd-treated cells (Figure 3e). Furthermore, immunofluorescence analysis revealed that COX-2 signals were stronger in the cytoplasm and perinuclear space of Cd-treated cells than in that of control cells (Figure 3f), which was consistent with other reports that COX-2 expressed in the perinuclear space in granulocytes^21^ and HaCaT cells.^22^

### Inhibition of COX-2 reverses Cd-induced autophagy

**Figure 4:**
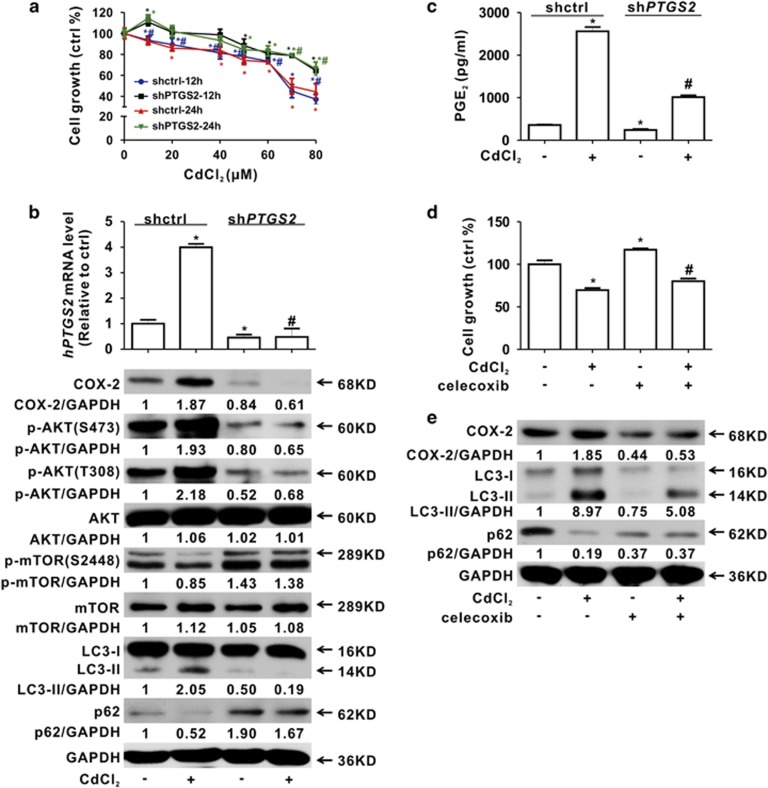
Cd-induced autophagy can be reversed by inhibition of COX-2. (**a**–**c**) Results of COX-2 knockdown using sh*PTGS2* cells: (**a**) alteration of cell growth (*significantly different from untreated control, ^#^significantly different from the same time in different cells, *P<*0.05); (**b**) COX-2 and autophagy and (**c**) production of PGE_2_. (**d** and **e**) Blocking COX-2 with celecoxib rescues 40 *μ*M Cd-induced cell growth inhibition with MTT assay (**d**) and autophagy with western blot analysis (**e**) in HEK cells for 12 h (*significantly different from untreated control, ^#^significantly different from Cd treatment. Data represent the mean±S.D.; *n*=3*, P<*0.05)

To elucidate whether COX-2 overexpression is required for Cd-induced autophagy, COX-2 was inhibited using gene knockdown or drugs. First, we found that cell growth was partially restored in HEK-sh*PTGS2* (COX-2 knockdown) cells compared with HEK-shctrl cells upon 10–80 *μ*M Cd exposure for 12 and 24 h (Figure 4a). Additionally, the expression level of *hMT1B* mRNA was also rescued in HEK-sh*PTGS2* cells in Supplementary Figure S3a. In Figure 4b, the increase of p-AKT and LC3-II, while the decrease of p-mTOR and p62 induced by Cd, was attenuated in HEK-sh*PTGS2* cells compared with the control cells, which was verified with the *PTGS2* small interference RNA (siRNA) transfection assay (Supplementary Figure S3b). Consistently, PGE_2_ was also decreased in HEK-sh*PTGS2* cells upon Cd exposure (Figure 4c). Next, celecoxib, a clinical COX-2 inhibitory drug, was used to treat HEK cells in combination with Cd. As shown in Figure 4d, celecoxib partially restored the cell growth inhibition caused by 40 *μ*M Cd. Celecoxib markedly inhibited COX-2 overexpression and subsequent autophagy in Cd-exposed HEK cells (Figure 4e). Additionally, we observed similar phenomena with other COX-2 inhibitors, CAY10404 and NS398 (Supplementary Figures S3c and d). These data demonstrated that Cd-induced autophagy was regulated by COX-2 *in vitro.*

### Cd-induced COX-2 overexpression depends on ER stress

**Figure 5:**
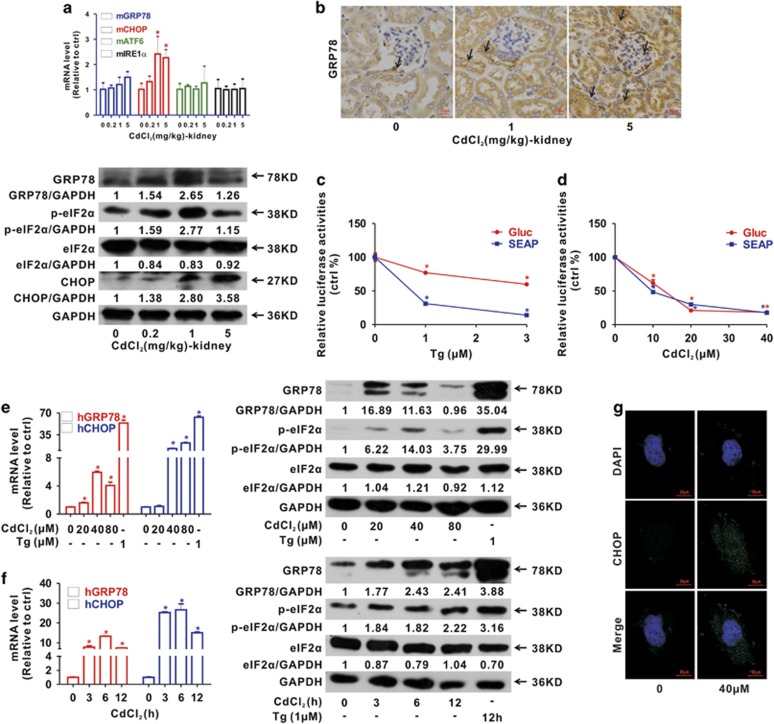
Cd induces ER stress both *in vivo* and *in vitro.* (**a**) Levels of mRNA (*mGRP78*, *mCHOP*, *mATF6* and *mIRE1α*) and proteins (GRP78, p-eIF2*α* and CHOP) related to ER stress caused by Cd for 7 days and (**b**) immunochemical analysis for GRP78 in the kidney tissues of Cd-exposed mice (magnification, × 400). Arrows indicate that the GRP78 signals increase in the glomerulus and tubules. Scale bar is 50 *μ*m. (**c** and **d**) ER stress reporter genes show ER stress occurs when HEK cells treated by Cd for 6 h. Tg used as a positive control. (**e** and **f**) ER stress related gene mRNA (*hGRP78* and *hCHOP*) and protein (GRP78 and p-eIF2*α*) levels in Cd-exposed HEK cells (**e**, concentration series of Cd; **f**, time series with 40 *μ*M Cd). (**g**) Confocal images for CHOP in the Cd-exposed HEK cells (40 *μ*M). Scale bar is 20 *μ*m (*significantly different from untreated control. Data represents the mean±S.D.; *n*=3*, P<*0.05)

We evaluated ER stress response upon Cd exposure both *in vivo* and *in vitro*. Cd significantly increased the mRNA level of *mGRP78* and *mCHOP* (two ER stress molecular markers) in a dose-dependent manner, but did not affect the *mATF6* or *mIRE1α* mRNA level in mice kidney (Figure 5a). Being consistent with these results, the GRP78 and p-eIF2*α* proteins were also induced by Cd in kidney tissues (Figure 5a). Moreover, immunohistochemical analysis confirmed that GRP78 was significantly increased in kidney tubules of Cd-exposed mice compared with control (Figure 5b and Supplementary Figure S2).

We examined the effect of Cd on ER stress by other means. Thapsigargin (Tg), an ER stress inducer, was used as a positive control. As shown in Figures 5c and d, Tg or Cd decreased the secretion of *Gaussia* luciferase (Gluc) and secreted alkaline phosphatase (SEAP) in a concentration-dependent manner in HEK-Fluc-Gluc and HEK-Fluc-SEAP cells. In addition, Cd elevated *hGRP78* and *hCHOP* mRNA and the GRP78 and p-eIF2*α* proteins in HEK cells (Figures 5e and f). Additionally, as depicted in Figure 5g, CHOP (CCAAT-enhancer-binding protein homologous protein) increased in Cd-exposed cells compared with control cells as detected using immunofluorescence analysis.

**Figure 6:**
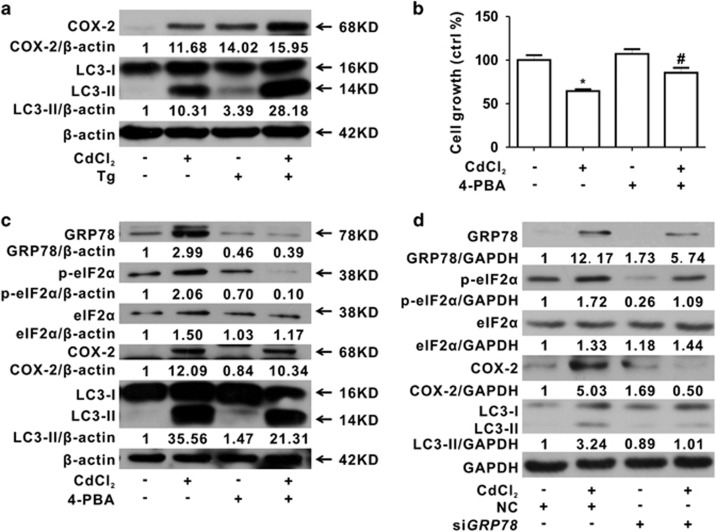
COX-2 induction in HEK cells by Cd requires ER stress. (**a**) 40 *μ*M Cd-induced autophagy is enhanced by Tg (1 *μ*M) in HEK cells. (**b** and **c**) 5 *μ*M 4-PBA treatment partially counteracts Cd-induced cell growth inhibition, ER stress and autophagy. (**d**) *GRP78* silencing also decreases Cd-induced ER stress and autophagy (*significantly different from untreated control, ^#^significantly different from Cd treatment. Data represents the mean±S.D.; *n*=3, *P<*0.05)

To elucidate whether Cd-induced ER stress is required for overexpression of COX-2, Tg was used to treat HEK cells combining with Cd. Tg significantly enhanced both the overexpression of COX-2 and autophagy induced by Cd, as indicated by an increased LC3-II (Figure 6a). 4-PBA, an ER stress inhibitor, counteracted the cell growth inhibition, COX-2 overexpression, ER stress and autophagy caused by Cd (Figures 6b and c). Moreover, when HEK cells were transfected with *GRP78* siRNA, followed by Cd exposure, the knockdown of *GRP78* or *CHOP* also significantly attenuated the Cd-induced COX-2 overexpression, ER stress and autophagy in HEK cells (Figure 6d and Supplementary Figure S4). Taken together, these results clearly indicated that ER stress is required for overexpression of COX-2 induced by Cd.

### Cd induces COX-2 overexpression via ATF4 transcriptional regulation

**Figure 7:**
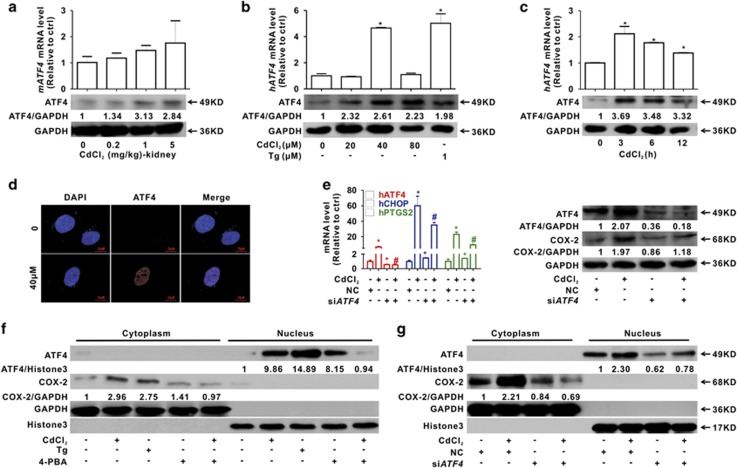
COX-2 induction by Cd is mediated by ATF4 transactivation. (**a**) Cd increases the levels of *mATF4* mRNA and ATF4 protein in kidney tissues. (**b** and **c**) Cd elevates *hATF4* mRNA and *ATF4* protein levels in HEK cells. Tg is used as a positive control (**b**, as a function of Cd concentrations for 12 h; **c**, as a function of time with 40 *μ*M Cd). (**d**) In HEK cells, Cd causes ATF4 translocation from the cytoplasm to the nucleus as detected using a confocal microscopy. Scale bar is 20 *μ*m. (**e**) *ATF4* silencing restores Cd-induced *PTGS2* mRNA and COX-2 protein overexpression. NC is the negative control of siRNA. (**f**) 4-PBA inhibits ATF4 translocation and COX-2 upregulation induced by Cd. (**g**) *ATF4* silencing also decreases Cd-induced ATF4 translocation and COX-2 upregulation (*significantly different from untreated control, ^#^significantly different from Cd treatment. Data represents the mean±S.D.; *n*=3, *P<*0.05)

To investigate the molecular mechanism of Cd-induced overexpression of COX-2 with ER stress, the transcription factor ATF4 was examined. In Figure 7a, Cd slightly increased *mATF4* mRNA, although there was no significant change compared with control, and ATF4 protein was elevated by Cd in kidney tissues. In addition, Cd increased the *hATF4* mRNA and ATF4 protein in HEK cells (Figures 7b and c). Immunofluorescence analysis revealed that ATF4 protein was translocated from the cytoplasm to the nucleus (Figure 7d). Furthermore, *ATF4* siRNA significantly inhibited the activation of ATF4 and COX-2 protein levels in HEK cells (Figure 7e). Finally, we found that ATF4 was translocated into the nucleus and COX-2 was transcriptionally activated upon Cd treatment, changes that were inhibited by blocking ER stress with 4-PBA, whereas ATF4 translocation and COX-2 expression were enhanced by activation of ER stress with Tg (Figure 7f). Interestingly, we found that, by itself, 4-PBA could increase ATF4 translocation into the nucleus, which might be due to the side effect of 4-PBA on cell signaling related to ATF4. We also observed that Cd-induced COX-2 was counteracted by the *ATF4* siRNA (Figure 7g). These results indicated that transcriptional activation of ATF4 was essential for COX-2 overexpression in Cd-treated HEK cells.

## Discussion

Our study provided evidence that Cd caused kidney injury and dysfunction with autophagy in mice. Cd also induced cell growth inhibition and autophagy in kidney cells, which supported previous studies.^10^ Next, we found that Cd-induced autophagy was associated with COX-2 overexpression both *in vivo* and *in vitro*. Further studies demonstrated that several COX-2 inhibitors, such as celecoxib, rescued the kidney from Cd-induced injury and autophagy, which was further confirmed by *PTGS2* RNAi protection against Cd damage in HEK cells. Additionally, ER stress, which was proved to be required for COX-2 overexpression in kidney tissues and cultured cells, occurred upon Cd treatment. Finally, our study demonstrated that the eIF2*α*–ATF4 pathway activated COX-2 transcription to regulate Cd-induced autophagy. We believe that this study has found a novel molecular mechanism of Cd-induced renal autophagy and injury, which is dependent on COX-2 overexpression regulated by ER stress.

The toxic heavy metal Cd is known to cause *itai-itai* disease, characterized by bone softening and kidney failure.^23^ More evidence shows that Cd exposure causes severe liver and kidney damage both *in vivo* and *in vitro*.^24, 25, 26^ Cd first accumulates in the liver after exposure.^27^ Most of the Cd is captured by MT proteins to form Cd/MT complexes that are gradually released into the blood and reabsorbed from the glomerular filtrate in the kidney.^27^ Then, the complexes degrade quickly to release the Cd, leading to renal injury.^28^ Our study showed that Cd exposure resulted in hepatomegaly, which was consistent with previous studies.^29, 30^ *mMTs* mRNA was induced by Cd both in livers and kidneys, which indicated that Cd had been transferred and released into the kidney which might result in renal damage. In line with these data, histological analysis showed glomerular shrinkage and tubular degeneration upon Cd exposure. We further examined serum renal function parameters. CRE is cleared from blood mainly by glomerular filtration as well as proximal tubular secretion within the kidney. A rise of serum CRE is observed only in the significantly injured kidney.^31^ BUN is another indicator of renal function. Once the kidney is damaged and dysfunctional, urea nitrogen is not filtered totally from the blood, resulting in BUN elevation.^32^ However, since urea is synthesized by liver, BUN will be lower than the normal level when the liver is severely damaged.^33^ A low BUN/CRE ratio indicates reduction of BUN reabsorption caused by kidney damage.^31^ In our study, Cd raised the serum CRE and BUN in the 0.2 and 1 mg/kg groups, indicating kidney filtration failure, but not in the 5 mg/kg group, which might have resulted from severe hepatomegaly and liver damage. The ratio of BUN/CRE decreased in all Cd groups, suggesting that Cd caused severe tubule damage and glomerular filtration failure. In support of our results, exposure of mice to 1.5 mg/kg Cd for 1 week also caused the histological alteration and dysfunction of kidney.^34^

We further found that Cd induced autophagy both *in vivo* and *in vitro*, which is consistent with a recent study showing that 0.3 mg/kg Cd induced autophagy in the kidney.^10^ Growing evidence indicates that autophagy may play a protective role against Cd-induced nephrotoxicity. An animal study showed that inhibition of autophagy with CQ and 3-methyladenine worsened ischemia–reperfusion-induced mice kidney injury and autophagy.^35^ *Atg5*-deficient mice had markedly affected kidney function and were more sensitive to kidney ischemic injury.^36^ Indeed, autophagy is either a survival or a death safeguard mechanism, which depends on the extent of stress. A review by Scarlatti *et al.*^37^ stated that once cells are severely damaged, autophagy can remove most of the organelles and kill the cells by a caspase-independent form of cell death, known as autophagy-dependent cell death. For examples, salivary Pa-4 cells treated with baflomycin A1 or CQ rescued the cell viability inhibition induced by H_2_O_2_.^38^ Caspase-8 inhibition could have the untoward effect of exacerbating cell death by activating the autophagic death pathway induced by Z-Val-Ala-Asp-fluoromethylketone (zVAD-FMK).^39^ In the present study, we found inhibition of autophagy with CQ would prevent the Cd-induced cell growth inhibition. Moreover, blocking autophagy upstream signaling, such as COX-2 and ER stress, also improved cell growth. We therefore deduced that autophagy induced by Cd might cause kidney cell growth inhibition and result in kidney failure.

The kinase mTOR is a major negative regulator of autophagy,^40^ which is the key effector in the PI3K–AKT–mTOR pathway.^41^ Autophagy can be activated via inhibition of the PI3K–AKT–mTOR pathway.^42^ However, inhibition of mTOR by some chemicals, such as rapa, will promote AKT phosphorylation by blocking the phosphorylation of insulin receptor substrate 1 and p70 ribosoma S6 kinase,^41, 43^ which are known to be a part of a negative feedback mechanism of PI3K–AKT signal transduction.^44^ Our results showed that Cd inhibited mTOR but actually increased the phosphorylation of AKT, suggesting that Cd inhibited mTOR which might not depend on AKT. Another possible explanation for Cd-induced increased AKT phosphorylation could be due to the loss of mTOR-mediated feedback inhibition on AKT, similar to rapa. Consistently, we discovered that COX-2 knockdown increased p-mTOR leading to a decrease of autophagy and p-AKT.

A previous study showed that Cd induced ER stress along with autophagy in mice kidney.^10^ In the present study, we also found that autophagy and ER stress were triggered in Cd-exposed kidney tissues and HEK cells. We confirmed that Cd induced ER stress in HEK cells using two ER stress reporter systems. Then, we demonstrated that an ER stress inhibitor counteracted Cd-induced autophagy and cell growth inhibition in HEK cells, which was verified by the *GRP78* siRNA transfection assay. Further, previous studies showed that activation of ER stress induces autophagy.^15, 45^ These studies provided evidence that ER stress was required for Cd-induced autophagy in the kidney. Moreover, Cd-induced COX-2 overexpression was attenuated by *GRP78* or *ATF4* silencing, which suggested that COX-2-mediated autophagy induced by Cd required ER stress in the kidney.

We found that Cd increased COX-2 and PGE_2_, along with autophagy and ER stress *in vitro*. Inhibition of COX-2 by celecoxib or *PTGS2* RNAi counteracted Cd-induced autophagy in the kidney. In support of these results, celecoxib has been proved to attenuate rodent renal injury induced by several other chemical or physical factors, such as cisplatin,^46^ cyclosporine^47^ or hypoxia.^48^ As long ago as 1997, celecoxib was developed^49^ and then used in the clinic.^50^ However, some case reports showed that long-time use or high doses of celecoxib raised the risk of renal injury in the elderly and in patients with pre-existing renal dysfunction, liver injury or heart diseases.^51^ Therefore, use of COX-2 inhibitors requires caution. More specific and safer COX-2 inhibitors or therapy strategy should be developed to guard against kidney injury.

Much work has studied the molecular mechanisms by which ER stress regulates autophagy. It has been reported that ER stress induces autophagy through activation of the RAF–MEK–ERK–MAPK cascade in melanoma.^52^ Qin *et al.*^53^ showed that autophagy triggered by ER stress depends on inhibition of the AKT–TSC–mTOR pathway. Furthermore, ER molecular chaperons and pathways, such as GRP78 protein and the IRE1*α*–JNK–Bcl-2, ATF6–XBP1–ATG and PERK–eIF2*α*–ATF4 pathways, are involved in ER stress-mediated autophagy.^54, 55, 56^ In the present study, we observed that Cd affected gene expression of *GRP78*, *ATF4* and *CHOP*, but not *IRE1α* or *ATF6,* in kidney tissues. Further, p-eIF2*α* and ATF4 proteins were activated by Cd both *in vivo* and *in vitro*. Cd-induced autophagy was counteracted by ER stress blocking. Therefore, it is likely that the eIF2*α*–ATF4 pathway regulates Cd-induced kidney autophagy and injury.

Specifically, the present study demonstrated that Cd promoted ATF4 translocation from the cytoplasm to the nucleus. Silencing of *ATF4* inhibited both its translocation and the COX-2 overexpression induced by Cd. Once translocated into the nucleus, ATF4 binds to the promoter region of *PTGS2* gene and promotes its transcription.^57^ These studies revealed a novel pathway by which Cd triggers autophagy via COX-2 overexpression, which is transcriptionally regulated by ATF4.

**Figure 8:**
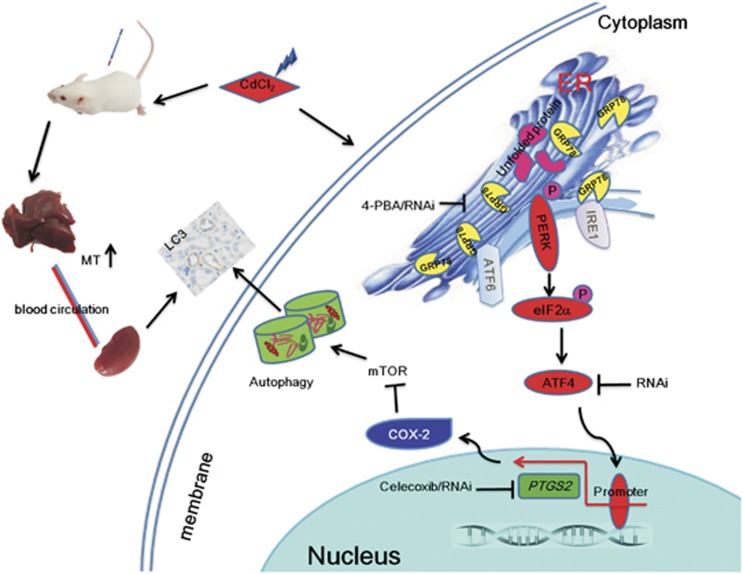
Cd induces kidney autophagy mediated by the eIF2*α*–ATF4 pathway in ER stress and its downstream molecule COX-2

In conclusion, the present study showed that Cd exposure resulted in kidney injury with autophagy. We demonstrated that Cd-induced autophagy was mediated by the eIF2*α*–ATF4 pathway and its downstream molecule COX-2, as illustrated in Figure 8. COX-2 has been proven to be a potential molecular marker of Cd-induced nephrotoxicity. The present study also suggests that safer COX-2 inhibitors or other therapy strategies should be developed for treatment of nephrotoxicity induced by xenobiotics.

## Materials and Methods

### Chemicals and reagents

The reagents used were as follows: cadmium chloride (CdCl_2_, purity>99%), CQ, rapa, 4-PBA and Tg (Sigma, St. Louis, MO, USA); NS398 and CAY10404 (Cayman, Ann Arbor, MI, USA) and celecoxib (Pfizer, New York, NY, USA).

### Animal exposure and sample collection

Adult male ICR mice (30–35 g, 10–12 weeks old) were randomly separated into control and three exposure groups (*n*=5–6) and housed in a ventilated animal room at 22±2 °C with a 12 h light/dark cycle. The mice were intraperitoneally injected at doses of 0.2, 1 or 5 mg/kg body weight CdCl_2_ every day for 1 week. The CdCl_2_ was dissolved in 0.9% physiological saline. In the control group, the mice were injected with 0.9% physiological saline. Twenty-four hours after the last injection, all mice were killed and serum, livers and kidneys were sampled for experimental purposes. The ratios of organ weight to body weight were calculated. Standard hematological renal functions were analyzed using a Cobas 8000 system (Roche, Basel, Switzerland) in Xiamen Zhongshan Hospital. All experiments were approved by the Animal Ethical Committee of Xiamen University and conducted in accordance with the guidelines of the National Institutes of Health.

### Histology and immunohistochemistry

After being embedded in paraffin blocks, kidney tissues were sectioned at 5 *μ*m thickness and the sections were placed on microscope slides. HE staining was performed on the slides, which were then observed and photographed with an optical microscope (TS-100, Nikon, Tokyo, Japan). For immunohistochemical analysis, the slides were dewaxed with xylene and rehydrated with graded ethanol. After incubation with 3% hydrogen peroxide to block endogenous peroxidase, the slides were incubated with 10% goat serum for 30 min to block nonspecific proteins. Then, the slides were incubated with specific primary antibodies at 4 °C overnight, followed by horseradish peroxidase-conjugated secondary antibody incubation for 30 min. Slides were then incubated with developing solution (peroxidase substrate containing diaminobenzidine) with hematoxylin counterstaining. Lastly, images were captured. Brown signals, showing immunoreactivity, were analyzed using Image Pro Plus 6.0. (Media Cybernetics,Bethesda, MD, USA).

### Cell culture

HEK cells were cultured in HyQ RPMI-1640 medium (Life, New York, NY, USA) with 10% fetal bovine serum. HEK293T cells were used for lentivirus packaging and were maintained in Dulbecco minimum essential medium with 10% fetal bovine serum. All culture media contained 1% (v/v) penicillin–streptomycin (Life). Cells were cultured in a humidified incubator with 5% CO_2_ at 37 °C.

### Cell growth inhibition

To determine cell growth inhibition, 8000 cells were plated in each well of 96-well plates and attached to the substratum for 24 h. The cells were exposed to various concentrations of Cd for 12 and 24 h. Untreated cells served as a control. Cell growth inhibition was determined using a 0.5 mg/ml (3-(4,5-dimethylthiazol-2-yl)-2,5-diphenyltetrazolium) bromide (MTT, Sigma) incubation for 4 h. The formazan product was then dissolved in 100 *μ*l lysis buffer. The plates were read against reagent blank at 570 nm using a microplate reader (Multiscan, Thermo, Waltham, MA, USA). Control cells were taken as 100% viability.

### Enzyme-linked immunosorbent assay (ELISA)

The production of PGE_2_ was measured using an ELISA kit from Cayman Chemical (Ann Arbor, MI, USA) according to the manufacturer's instruction.

### Construction of reporter cell lines

The gene expression lentiviral vectors pLv-EF1*α*-Gluc-IRES-Puro, pLv-EF1*α*-SEAP-IRES-Puro and plv-EF1*α*-Firefly luciferase (Fluc)-IRES-Bsd were gifts from Dr. Lei Guo (Food and Drug Administration, USA). Two stable cell lines (HEK-Fluc-Gluc and HEK-Fluc-SEAP) were constructed as described previously.^58^ Briefly, lentiviral stocks were produced by transfection of HEK293T cells with a mixture of a lentiviral vector and viral packaging plasmids (pMDL-G, pVSV-G and pRSV-REV) in the ratio of 10 : 5 : 2 : 3, using Lipofectamine 2000 (Life). To construct a stable Fluc cell line, plv-EF1*α*-Fluc-IRES-Bsd plasmid was infected into HEK cells with lentivirus and screened with 10 *μ*g/ml blasticidin. Then, the cells containing Fluc gene were infected with lentivirus carrying Gluc or SEAP. The cells were screened for successful transfection with 0.8 *μ*g/ml puromycin.

### Construction of COX-2 knockdown cell lines

Two stable cell lines were generated as described previously.^59^ Briefly, using the DNA Transfection Reagent X-tremeGENE HP (Roche), the lentiviral vectors described above, and viral packaging plasmids (pCMV-delta 8.9 and VSVG), were co-transfected into HEK293T cells to produce lentiviral stocks. HEK cells were infected with lentivirus carrying pLKO-*PTGS2*-shRNA recombinant plasmid. Then, the cells were selected with 0.8 *μ*g/ml puromycin. The established *PTGS2* knockdown HEK cells were identified and then named HEK-sh*PTGS2,* and the corresponding control cells were called HEK-shctrl.

### siRNA transfection

*PTGS2*, *GRP78* and *ATF4* siRNA and a negative control (NC) were designed and synthesized by RiboBio Company (Guangzhou, China). HEK cells were seeded at close to 30% confluence without any antibiotics. Twenty-four hours later, specific siRNA or NC (50 nM) was added into cells with Lipofectamine 2000 according to the manufacturer's instructions.

### Luciferase activity assay

Gluc activity was measured by adding 50 *μ*l Gluc assay reagent (Nanolight Technology, Pinetop, AZ, USA) to 20 *μ*l of the Gluc sample and analyzing with a GLOMAX 96 microporous plate light reader (Promega, Madison, WI, USA) to determine the bioluminescence. Fluc activity was similarly analyzed: 20 *μ*l luciferase cell lysis buffer (NEB, Ipswich, MA, USA) was added to cellular extract, and then 100 *μ*l of the Fluc assay reagent (Lulong, Xiamen, China) was added to the cell lysate and read immediately.

### SEAP assay

SEAP was detected by using the Phospha-Light EXP assay kit (Life) according to the manufacturer's manuals. Briefly, an aliquot of 50 *μ*l cell-free medium was mixed with 50 *μ*l of 1 × dilution buffer, heated at 65 °C for 30 min, and then cooled on ice. Fifty microliters of the diluted sample was incubated in a well of a white 96-well assay plate with 50 *μ*l assay buffer for 5 min. Next, 50 *μ*l of reaction buffer was added and incubated for 20 min. The chemiluminescence was determined with the GLOMAX 96 microporous plate light reader.

### Quantitative real-time PCR (qRT-PCR)

The total RNA was extracted from the tissues or cells using TRIzol reagent (Life) according to the manufacturer's protocol. One microgram of total RNA was reverse transcribed into cDNA using TaKaRa reverse transcription reagents (TaKaRa, Otsu, Japan). The expression level of target genes was measured by qRT-PCR using the SYBR Green I kit (TaKaRa) on a Bio-Rad CFX96 Real-Time PCR Detection System (Bio-Rad, Hercules, CA, USA). The amplification conditions were: initial denaturation at 95 °C for 30 s, 42 cycles of 95 °C for 5 s and 60 °C for 34 s. *hACTB* or *mGAPDH* mRNA was used as the reference for human or mouse gene expression, respectively. The primers' sequences were listed in Supplementary Table S1.

### Western blot analysis

The kidney tissues were homogenized and lysed in RIPA buffer (Beyotime, Nantong, China) with protease inhibitors. The cells were lysed in whole-cell lysate buffer with phosphatase and protease inhibitors (Beyotime). Proteins were separated by sodium dodecyl sulfate-polyacrylamide gel electrophoresis and transferred to a polyvinylidene difluoride membrane (Millipore, Boston, MA, USA). The membranes were incubated with primary antibodies including COX-2 (Abcam, Cambridge, UK; dilution 1 : 1000), HistoneH3 (Beyotime; dilution 1 : 1000), *β*-actin (ProteinTech, Chicago, IL, USA; dilution 1 : 3000), GAPDH (Kangchen, Shanghai, China; dilution 1 : 5000), p-AKT(Ser473), p-AKT(Thr308), p-mTOR(Ser2448), mTOR, GRP78, p-eIF2*α*, ATF4, CHOP, LC3 and p62 (CST, Danvers, MA, USA; 1 : 1000) overnight at 4 °C, followed by a horseradish peroxidase-conjugated IgG secondary antibody (dilution 1 : 3000) for 1 h at room temperature. Photographs were captured and the relative density of blotting was quantified using Image J software (NIH, Bethesda, MD, USA).

### Immunofluorescence assay

HEK cells were grown in six-well plates with or without 40 *μ*M Cd for 12 h, and then fixed in 4% paraformaldehyde (pH 7.2–7.4) for 20 min. The samples were incubated in PBS plus 0.3% Triton X-100 for 5 min on ice. After blocking with 1% BSA for 30 min, the cells were incubated overnight at 4 °C with COX-2, ATF4, CHOP or LC3 antibody (dilution 1 : 200). After washing, the samples were incubated with goat anti-mouse/rabbit IgG secondary antibody (Alexa Fluor 594, Life) for 1 h in the dark. Next, the cells were counterstained with DAPI (Beyotime). Finally, they were imaged using a confocal microscope (Carl Zeiss, Jena, Germany).

### Statistics

All results were expressed as the mean±standard deviation (S.D.). The statistical analyses were conducted using an unpaired Student's *t*-test with SPSS 18.0 software, and *P*<0.05 (two tailed) was used as the criterion for significant difference.

## Abbreviations

4-PBA: 4-phenylbutyrate
AKT: RAC-*α* serine/threonine-protein kinase
ATF4: activating transcription factor 4
ATF6: activating transcription factor 6
ATG: autophagy-related gene
BUN: blood urea nitrogen
Cd: cadmium
CHOP: CCAAT-enhancer-binding protein homologous protein
COX-2: cyclooxygenase-2
CQ: chloroquine
CRE: creatinine
eIF2*α*: eukaryotic initiation factor 2*α*-subunit
ELISA: enzyme-linked immunosorbent assay
ER: endoplasmic reticulum
ERK: extracellular signal-regulated kinase
Fluc: firefly luciferase
Gluc: *Gaussia* luciferase
GRP78: glucose-regulated protein 78
HE: hematoxylin and eosin
HEK: human embryonic kidney
IC_50_: 50% growth inhibition concentration
IRE1*α*: inositol-requiring enzyme 1*α*
JNK: Jun N-terminal kinase
LC3: microtubule-associated protein 1 light chain 3
MAPK: mitogen-activated protein kinase
MEK: mitogen-activated protein/ERK kinase
mTOR: mammalian target of rapamycin
MT: metallothionein
MTT: (3-(4,5-dimethylthiazol-2-yl)-2,5-diphenyltetrazolium) bromide
NC: negative control
PERK: protein kinase RNA-like endoplasmic reticulum kinase
PGE_2_: prostaglandin E_2_
PTGS2: prostaglandin-endoperoxide synthase 2
qRT-PCR: quantitative real-time PCR
rapa: rapamycin
RNAi: RNA interference
SEAP: secreted alkaline phosphatase
siRNA: small interference RNA
Tg: thapsigargin
TSC: tuberous sclerosis complex
UPR: unfolded protein response
zVAD-FMK: Z-Val-Ala-Asp-fluoromethylketone
