# Supplementary material for: Endoplasmic reticulum stress eIF2α–ATF4 pathway-mediated cyclooxygenase-2 induction regulates cadmium-induced autophagy in kidney
Source: Cell Death Dis. 2016 Jun 2;7(6):e2251–. doi: 10.1038/cddis.2016.78 (PMC5143407; doi:10.1038/cddis.2016.78)
Supplement: Supplementary Information [file cddis201678x1.docx]

**Supplement files:**

Table S1 Sequences of the primers for qRT-PCR.

| **Genes** | **Forward primers** | **Reverse primers** |
| --- | --- | --- |
| ***hACTB*** | 5′-CACCAGGGCGTGATGGT-3′ | 5′-CTCAAACATGATCTGGGTCAT-3′ |
| ***hPTGS2*** | 5’-CAGCCATACAGCAAATCCTTG-3’ | 5’-CAAATGTGATCTGGATGTCAAC-3’ |
| ***hGRP78*** | 5′-CATCACGCCGTCCTATGTCG-3′ | 5′-CGTCAAAGACCGTGTTCTCG-3′ |
| ***hATF4*** | 5′-CTGAGCAGCGAGGTGTTGGT-3′ | 5′-ACAGCCAGCCATTCGGAGGA-3′ |
| ***hCHOP*** | 5′-TTCTCTGGCTTGGCTGACTGA-3′ | 5′-TGGTCTTCCTCCTCTTCCTCCT-3′ |
| ***hPERK*** | 5′-TCCAGCCCAGTTCACCAAAGG-3′ | 5′-AACCGTCTGCTCTTCCTCATCC-3′ |
| ***hMT1B*** | 5'-GGCTTGTCTTGGCTCCAAATG-3' | 5′-GCAAACCGGTCAGGGTAGTT-3′ |
|  |  |  |
| ***mGADPH*** | 5'-TTGATGGCAACAATCTCCAC-3' | 5'-CGTCCCGTAGACAAAATGGT-3' |
| ***mPTGS2*** | 5'-CAGATGACTGCCCAACTCCC- 3' | 5'-GAACCCAGGTCCTCGCTTAT- 3' |
| ***mGRP78*** | 5'-AACCAACTCACGTCCAACCC-3' | 5'-TCTTTCCCAAATACGCCTCAG-3' |
| ***mATF4*** | 5'-GCCAAGCACTTGAAACCTCA-3' | 5'-CTCCAACATCCAATCTGTCCC-3' |
| ***mCHOP*** | 5'-CCTGCCTTTCACCTTGGAG-3' | 5'-AGCGAGGGCTTTGGGATGT-3' |
| ***mIRE1α*** | 5'-CCAGGATGTAAGTGACCGAATAG-3' | 5'-GGAAGCGGGAAGTGAAGTAG-3' |
| ***mATF6*** | 5'-CAGACACTACCAGCCCTTATGC-3' | 5'-TTGTAGAACAGGTTTAGTCACGG-3' |
| ***mMT1*** | 5'-CTCTAAGCGTCACCACGACTTC-3' | 5'-CGTCACATCAGGCACAGCAC-3' |
| ***mMT2*** | 5'-CGCTCCTAGAACTCTTCAAACC-3' | 5'-GCACTTCGCACAGCCCAC-3' |
|  |  |  |


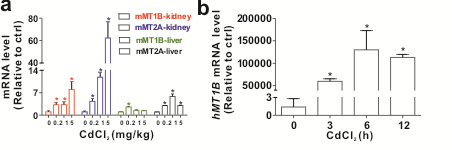


Figure S1 MT genes are induced by Cd in the kidney and liver tissues and HEK cell. (a) As a function of Cd concentrations for seven days and (b) time with 40 μM Cd. (* Significantly different from untreated control. Data represents the mean ± S.D., n=3, *P<*0.05).


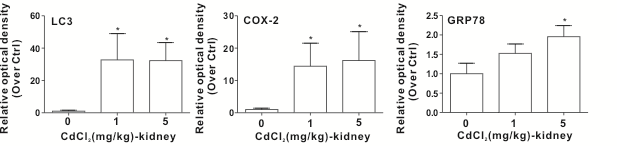


Figure S2 Relative optical densities of immunohistochemical staining of LC3 (Figure 2b), COX-2 (Figure 3b), and GRP78 (Figure 5b) in Cd-treated kidney. (* Significantly different from untreated control. Data represents the mean ± S.D., n=3, *P<*0.05)


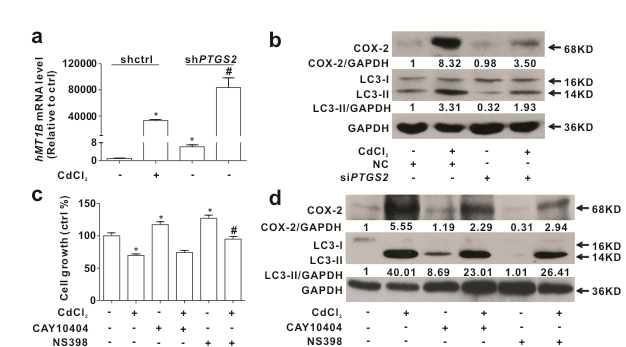


Figure S3 (a) *hMT1B* induction by Cd is augmented by knocking down COX-2 using sh*PTGS2* cells (* Significantly different from untreated shctrl cell, ^~~#~~^ Significantly different from Cd treatment, *P<*0.05). (b) Knockdown COX-2 with siRNA decreases Cd-induced autophagy in HEK cells (40 μM). (c, d) Similar to celecoxib (20 μM), CAY10404 (20 μM) and NS398 (20 μM) partially rescue Cd-induced cytotoxicity (c) and autophagy (d) (* Significantly different from untreated control, ^#^ significantly different from Cd treatment, n=3, *P<*0.05). Data represents the mean ± S.D., n=3.


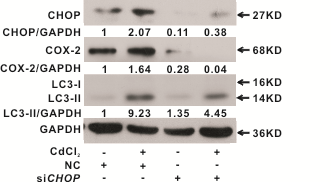


Figure S4 Knocking down CHOP with siRNA inhibits 40 μM Cd-triggered COX-2 overexpression and autophagy in HEK cells.
